# Vasopressin and oxytocin expression in hypothalamic supraoptic nucleus and plasma electrolytes changes in water-deprived male *Meriones libycus*

**DOI:** 10.1080/19768354.2021.1986130

**Published:** 2021-10-10

**Authors:** Lydia Boumansour, Nadir Benhafri, Gilles Guillon, Maithe Corbani, Hanane Touati, Aicha Dekar-Madoui, Saliha Ouali-Hassenaoui

**Affiliations:** a Faculty of Biological Sciences, Laboratory of Biology and Physiology of Organisms, Neurobiology, University of Sciences and Technology Houari Boumediene (USTHB), Algiers, Algeria; b Université de Montpellier, CNRS, ISERM, Institut de Génomique Fonctionnelle, Montpellier, France

**Keywords:** Desert rodent, dehydration, plasmatic osmolality, supraoptic nucleus, vasopressin

## Abstract

In mammals, plasmatic osmolality needs to be stable, and it is highly related to the hydric state of the animals which depends on the activity of the hypothalamic neurohypophysial system and more particularly by vasopressin secretion. Meriones, a desert rodent, can survive even without drinking for more than one month. The mechanism(s) by which they survive under these conditions remains poorly understood. In this study, we examine the water’s deprivation consequences on the: (1) anatomy, morphology, and physiology of the hypothalamic supraoptic nucleus, (2) body mass and plasma electrolytes changes in male desert rodents ‘*Meriones libycus*’ subjected to water deprivation for 30 days. The effect of water deprivation was evaluated on the structural and cellular organization of the supraoptic nucleus by morphological observations and immunohistochemical approaches, allowing the labeling of AVP but also oxytocin. Our finding demonstrated that upon water deprivation (1) the body weight decreased and reached a plateau after a month of water restriction. (2) The plasmatic osmolality began to decrease and return to values similar to control animals at day 30. (3) The SON, both in hydrated and water-deprived animals, is highly developed.(4) The AVP labeling in the SON increased upon dehydration at variance with OT. These changes observed in body mass and plasma osmolality reveal an important adaptive process of male Meriones in response to prolonged water deprivation. Overall, this animal represents an interesting model for the study of water body homeostasis and the mechanisms underlying the survival of desert rodents to xeric environments.

## 1. Introduction

In mammals, the regulation of plasmatic osmolality is an essential physiological mechanism for normal development (Hussy et al. 2000). Regulation of the osmolality of extracellular fluids is achieved by balancing the intake and excretion of salts and water (Louden 2012). The neuroendocrine hypothalamo-neurohypophysial system responsible for this physiological regulation consists of neurons located in the paraventricular (PVN) and supraoptic nuclei (SON). These neurons (magnocellular neurons of the SON and those of the PVN nuclei) secrete two main neuropeptides: arginine vasopressin (AVP) and oxytocin (OT). These neuropeptides are stored in the neuron cell bodies, transported to the pituitary, and released in the general circulation upon physiological stimulation like dehydration. According to a large and recent literature, these neuropeptides have been shown to affect water reabsorption, arterial blood pressure, glucose homeostasis, ACTH secretion (Manning et al. 2012). One of the most important functions of AVP is to maintain homeostasis (e.g. water retention, blood pressure, circadian rhythms and temperature regulation, arousal activation, and memory), while OT is involved in the maintenance of the social group and/or species (e.g. ovulation, parturition, lactation, sexual behavior, and social interactions) but also suppression of food intake (Finger 2011; Benarroch 2013; Ludwig et al. 2017).

Small mammals, such as desert rodents can survive for long periods in extreme environmental conditions without free water and resist the effects of dehydration by obtaining preformed water from food and metabolic water (Degen 1997). The main avenues for water loss are respiration, urine, feces, and thermoregulatory mechanisms, such as sweating, salivation, and evaporative cooling (Schwimmer and Haim 2009). Many homeostatic mechanisms and physiological adaptations to deserts have been characterized in several rodents like Gerboa, Pocket mice, Meriones, Jerbils (Ghobrial and Nour 1975; Baddouri et al. 1987; Baddouri and Quyou 1991; Ouali and Bensalem 1996). Interestingly, Meriones are characterized by their resistance to long periods of thirst and have a particular ability to support prolonged dehydration for periods up to three months (Laalaoui et al. 2001; Elgot et al. 2009).

It has been suggested also that electrolytes may be considered as important markers of dehydration (Cheuvront and Kenefick 2011). Interestingly, in humans and traditional mammalian models, the response to severe acute dehydration leading to severe electrolyte imbalance is poorly documented. In order to survive despite prolonged dehydration states, the main challenge for desert animals is to maintain the electrolyte gradients required for proper function (MacManes 2017).

In the present study, we investigated the effects of prolonged water deprivation (WD) on the morphological and the cellular organization of the SON as well as on plasma electrolytes and osmolality changes, and hematocrit; which is an indicator of plasma volume (Kutscher 1968) in male desert rodent *Meriones libycus.* Furthermore, using immunohistochemicals approaches, we evaluated AVP and OT expression in magnocellular neurons (MCNs) of the SON to better understand how these mammalian species adapt their AVP level of production in response to their arid environment.

## 2. Materials and methods

### 2.1. Animals

Only the minimum number of animals necessary to produce reliable scientific data was used. Experiments were carried out in male *Meriones libycus (Gerbillidae)*, a granivorous rodent captured in December around the desert region of Béni Abbes (Southwest of Algeria, with hot and extremely dry desert climate).

Fifty-six (56) wild adult males *Meriones libycus* (Body weight (BW): 80–100 g) (control *n* = 28, WD *n* = 28) were housed singly in stainless-steel cages. The colony room was maintained at a constant temperature (25 ± 1°C) under 12:12 h light–dark cycle, with free access to food (standard dried rat pellets) and tap water until the initiation of water deprivation protocol. Animals were kept water deprived for 30 days. Body mass and plasmatic electrolytes were measured on days 7, 14, 21 and 30. All experiments and animal care procedures were designed in accordance with the European guidelines on the ethical use of animals and have been approved by the ethical local Committee and the ‘Association Algérienne des Sciences en Expérimentation Animale (AASEA)’ (http://www.aasea.asso.dz/).

### 2.2. Slice preparation

Meriones were deeply anaesthetized (pentobarbital, 120 mg/100 g BW by i.p. injection) then perfused transcardially with PF 4% in phosphate buffer (0.1 M, pH 7.4). The Brains were carefully removed and post-fixed in the same fixative for 24 h at 4°C. The tissues collected were rinsed in Phosphate Buffer Saline (PBS) to wash out the fixative, dehydrated in graded ethanol solutions (70–100%), and embedded in pure paraffin. Frontal sections of the hypothalamus (10 μm) were realized with a microtome. Sections were performed throughout the SON. The immunolabeling was performed on selected sections in the median part of the nucleus where the cell density is maximal.

### 2.3. Immunohistochemistry

The microtome sections were mounted, deparaffinized, and rehydrated. The slices were then incubated in a moist chamber for 48 h at 4°C with one or two primary antibodies (Anti-Neurophysin 2/NP-AVP mouse IgG, 1:500, Millipore, USA, Anti-OT rabbit IgG, 1:4000, produced by G. Alonso, Montpellier, France) (Alonso et al. 2005).

After rinsing in PBS, sections were incubated for 1 h at room temperature with the corresponding secondary antibodies conjugated with Cy3 (donkey anti-rabbit IgG, Jackson ImmunoResearchLaboratories, USA, 1:2000) or Alexa Fluor 488 (goat anti-mouse IgG, Invitrogen Molecular Probes, USA, 1:2000). The antibodies were diluted in PBS containing 2% Bovine Serum Albumin (BSA) and 0.1% Triton X-100. After rinsing in PBS, sections were incubated for 30 min in DAPI (1:1000) (4′,6-diamidino-2-phenylindole) a nuclear counterstain for fluorescence microscopy.

Labeled sections were rinsed in PBS, mounted in Mowiol, and observed under Zeiss Axio Imager 2 fluorescence microscope with Apotome (IGF Montpellier, France).

The specificity of the vasopressin, oxytocin and commercial antibodies has been assessed by absorption tests. Additional negative and positive controls were applied. This allowed us to confirm the validity of the staining pattern and to exclude experimental artifacts.

### 2.4. Immunolabeling quantification

Quantitative analysis was performed on series of 10–12 sections per animal passing through the middle portions of the entire SON (i.e. the largest SON areas). The analysis was performed on three animals for each group (control and WD groups). The optical density (OD) of AVP and OT immunoreactivity was quantified using NIH Image J software. The OD value resulted from the difference between the staining intensity in the SON and the background intensity. Furthermore, regarding the distribution of AVP and OT neurons in the SON a semi-quantitative analysis was assessed on 5–10 sections per animal with a total of 75 images for controls and WD animals.

### 2.5. Plasma assays

Blood samples were collected from the infra-orbital sinus in plastic tubes containing heparin and centrifuged (3000*g* for 15 min). Plasma sodium and potassium concentration ([Na+] [K+]) were measured immediately thereafter using an ion-specific electrode (Easylyte^®^). The osmolality of each sample was measured using an Osmometer (Loeser type 6M). Other blood samples were collected in heparinized hematocrit capillary tubes. These samples were centrifuged at 1500*g* for 10 min at 4°C, and the hematocrit was determined directly (Hematokif-210).

### 2.6. Statistical analysis

Quantitative results are expressed as means ± S.E.M. We compared WD groups and controls using one-way analysis of variance (ANOVA) after an initial *F*-test for uniformity of variance. When differences were noted, *Post hoc* Scheffe test analysis was used. Differences were considered significant when *p*-value of <.05 (*); *p* < .01 (**); or *p* < .001 (***). Statistical analyses were performed with GraphPad Prism 6.0 (GraphPad software).

## 3. Results

### 3.1. Changes in body weight after water deprivation

**Figure 1.**
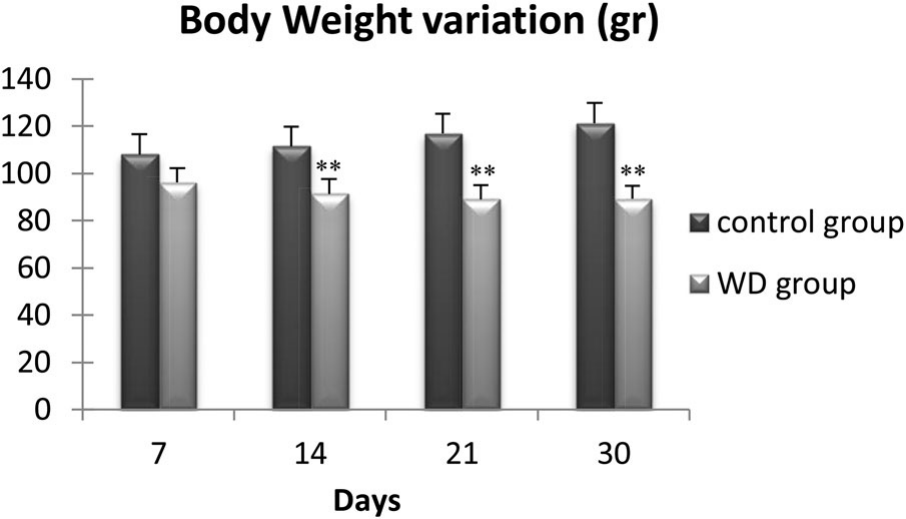
Evolution of body weight of control and water-deprived Meriones (WD) measured at days 7, 14, 21 and 30. Data are expressed as means ± S.E.M. (*) *p* < .05.

We measured the body mass of 56 wild adult males at days 7, 14, 21, and 30 (Figure 1). During the first thre weeks of water deprivation, we observed a progressive decrease of the Meriones body weight, which became significant only after two weeks of water restriction. Then, the body weight remained stable up to the 30th day. At this moment, WD Meriones lost 16.3 ± 8.1% of their body weight (*n* = 7).

### 3.2. Hematocrit and plasma osmolality

Both the hematocrit and the plasma osmolality provide reliable information about the body water content of an animal. We measured the hematocrit index and plasmatic osmolality of wild adult males Meriones at days 7, 14, 21, and 30.

**Figure 2.**
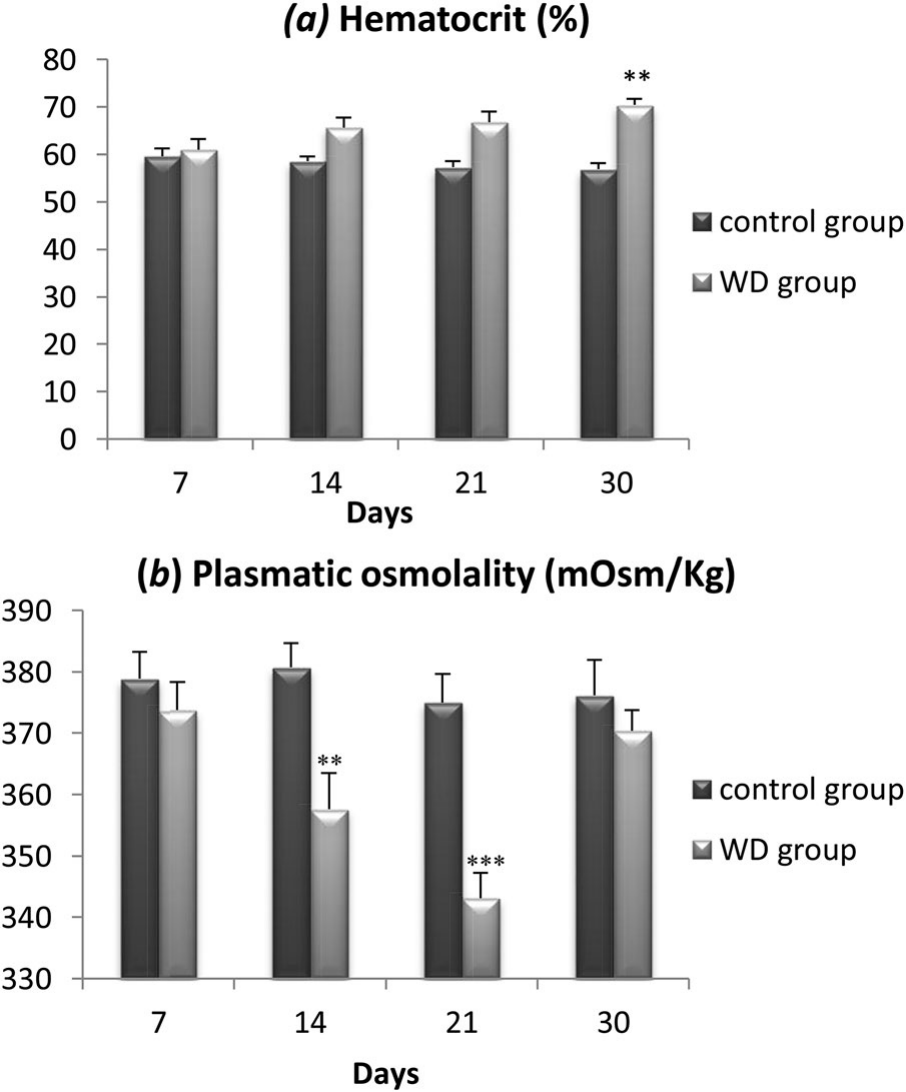
Evolution of Hematocrit (a) and plasmatic osmolality (b) in the controls and water-deprived Meriones (WD) measured at days 7, 14, 21 and 30. Data are expressed as means ± S.E.M. (*) *p* < .05.

Regarding the hematocrit index (Figure 2(a)) there is no significant difference between controls and WD Meriones during the first three weeks of water privation. However, after 30 days of water deprivation, the hematocrit index increased significantly by 12.14 ± 2.4% (*p* < .001).

For plasmatic osmolality (Figure 2(b)), there is no significant difference between controls and WD Meriones during the first week of water restriction. However, during the two next weeks, a significant decrease of 33.7 ± 8.2% was observed in WD animals compared to controls (*p* < .01). Interestingly, we observed a normalization of this parameter after a month of water deprivation and the plasmatic osmolality returned to control values despite a continuous water privation.

### 3.3. Electrolyte (Na^+^, K^+^) contents

**Figure 3.**
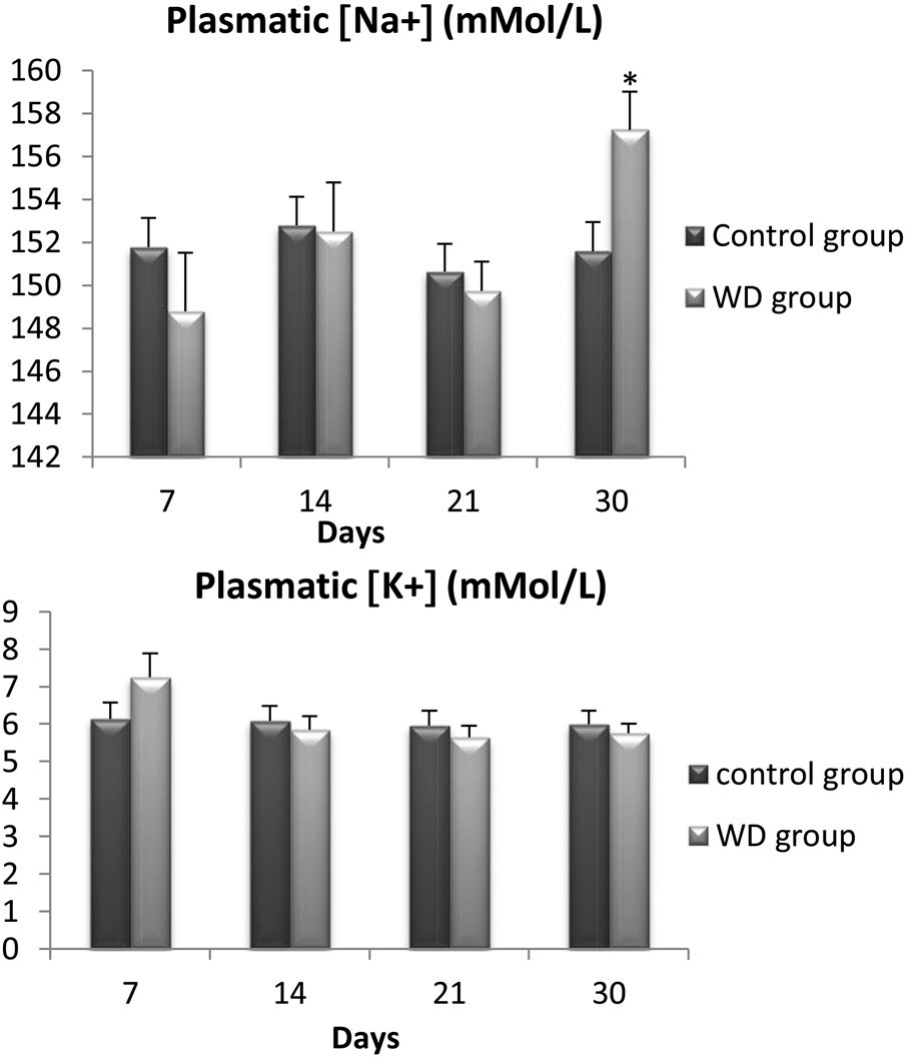
Evolution of plasmatic concentration of electrolyte (Na+, K+) in control and water-deprived Meriones measured at days 7, 14, 21 and 30. Data are expressed as means ± S.E.M. (*) *p* < .05.

We also measured levels of serum sodium and potassium for the same 56 wild adult males *Meriones libycus* at days 7, 14, 21, and 30. For sodium (Figure 3(a)), there was only a significant difference between WD Meriones and controls on the 30th day of water restriction (*p* < .05). However, no significant difference was observed for potassium between WD Meriones and controls (Figure 3(b)).

### 3.4. AVP and OT neurons distribution in the SON of Meriones

**Figure 4.**
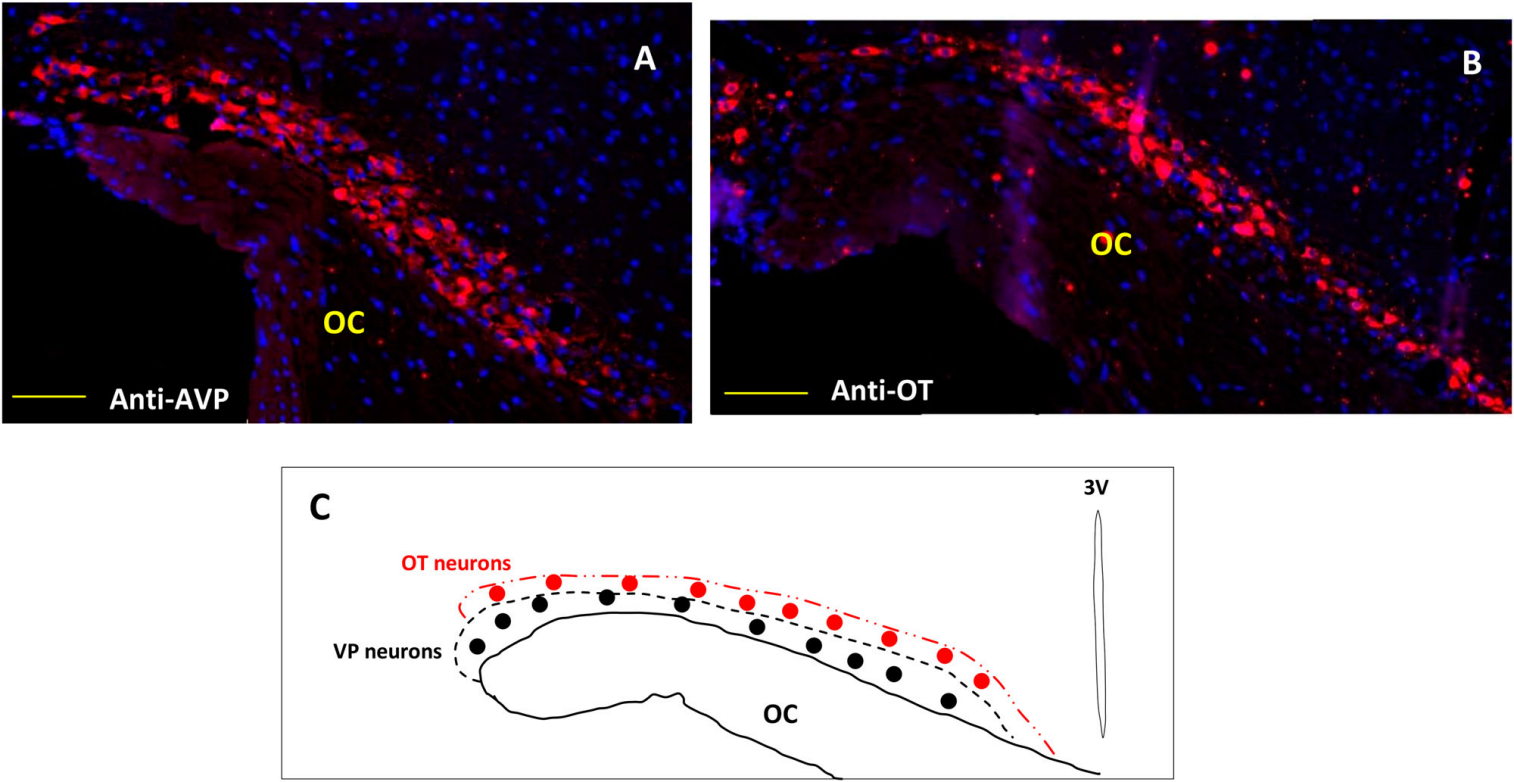
Immunofluorescent micrographs of frontal sections through the hypothalamus of *Meriones libycus* showing distribution of (A) magnocellular vasopressin (AVP, red) and (B) oxytocin (OT, red) neurons and nuclei (DAPI, blue) in the supraoptic nucleus (SON). The upper schematic illustrates the distribution of VP neurons (black dots) and OT neurons (red dots). OC: optic chiasm. Scale bar: 200 µm.

Different tests and positive or negative controls were assessed to validate the specificity of both the AVP and OT labeling (data not shown**)**. In comparison with laboratory rodents and other mammals, the hypothalamic magnocellular nucleus (SON) of the desert rodent *Meriones libycus* is highly developed; it has a large medio-lateral extension in the dorso-lateral part of the optic chiasm (OC). Both antibodies (anti-AVP and anti-OT) were used to label the whole population of SON neurons. In all SON sections, AVP and OT positive neurons were detected (Figure 4(A,B)). Strong immunolabeling characterized their round to ovoid shaped somata. The AVP neurons are mostly located in the lateral and ventral part of this nucleus, while the OT positive neurons are essentially localized in the dorso-lateral part of the SON (Figure 4(C)). Indeed, in the SON of Meriones most of the visible AVP-containing cells showed OT immunofluorescence, a semi-quantitative evaluation, on frontal sections revealed that immunoreactivity coexisted in about 37% of magnocellular neurons.

### 3.5. Effect of WD on AVP and OT expression in SON

Neurophysin 2 (NPII) (or vasopressin associated neurophysin NP-AVP) is a protein co-expressed with vasopressin and a carrier of peptide hormones Vasopressin (AVP). We used immunofluorescence to quantify the expression of NP-AVP.

**Figure 5.**
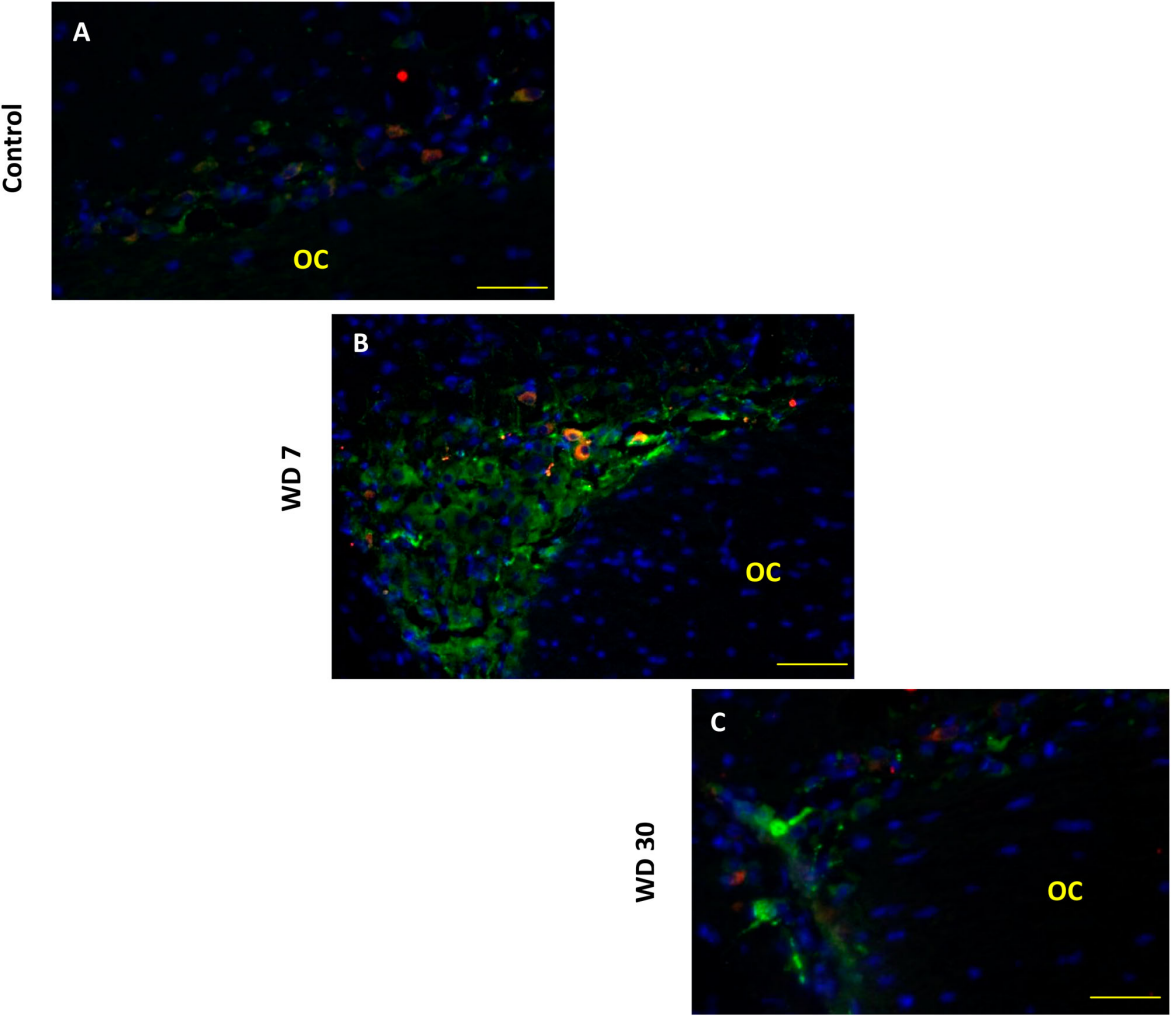
Double immunofluorescence staining in the frontal sections through the hypothalamus of *Meriones libycus* shows vasopressinergic (NP/AVP, green) and oxytocinergic (OT, red) neurons and nuclei (DAPI, blue) of the SON in control group (A), 7 days WD group (B) and 30 days WD group (C). NP/AVP colocalizes with OT (orange). OC: optic chiasm. Scale bar: 100µm

**Figure 6.**
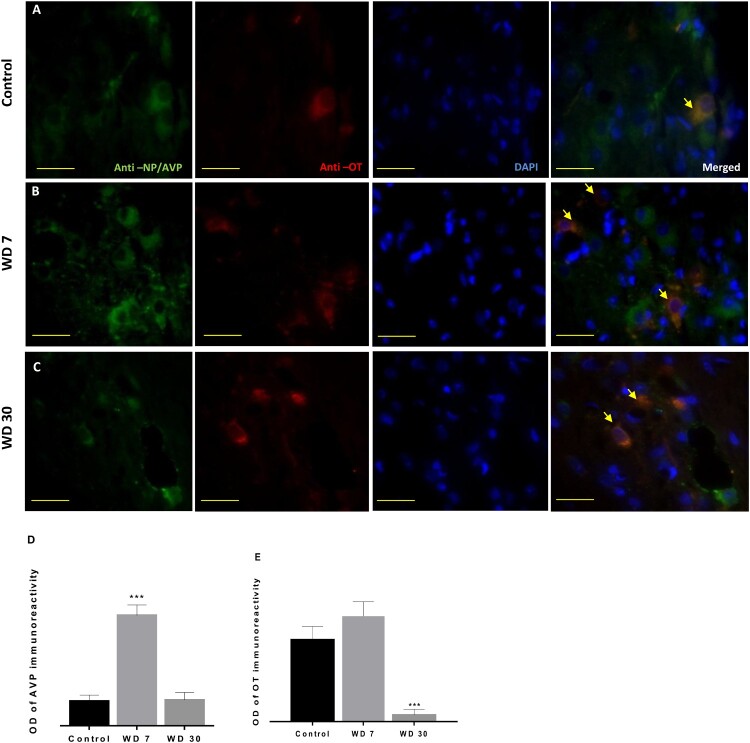
Double immunofluorescence shows vasopressinergic (NP/AVP, green) and oxytocinergic (OT, red) neurons in the SON of *Meriones libycus* in control group (A), 7 days WD group (B) and 30 days WD group (C). It also reveals many neurons showing immunofluorescence for both peptides (orange) in their somata (arrows). Histograms show the OD of immunoreactivity of AVP (D) and OT (E) in the magnocellular neurons of the SON in different groups. Values are mean ± SEM. OD: optical density. Scale bar: 50 µm.

Compared to controls, the WD animals of seven days showed a significant increase in NP-AVP immunoreactivity (Figure 5(A,B) and Figure 6(A, B and D)). However, after four weeks of water deprivation, the intensity of cytoplasmic NP-AVP staining goes back to the same level as control animals (Figure 5(C) and Figure 6(C,D)).

On the other hand, in the anti-OT immunolabeling after seven days of water deprivation, we observed a slight non-significant increase (Figure 5(A, B, and C) and Figure 6(A,B)). By contrast, in the 30 days WD group, a highly significant decrease of OT immunoreactivity was detected (Figure 6(E)).

## 4. Discussion

Desert rodents occupy habitats that present extraordinary demands, and they take up these challenges with evident success (Walsberg 2000). They display physiological features that favor body water conservation, such as efficient kidney function, low fecal water content, and comparatively lower evaporative water loss (Bozinovic and Gallardo 2006).

### 5.1. Body mass, plasma electrolytes, and hematocrit

In this study, we found that water deprivation leads to a decrease in body mass. This loss of weight was similar to other desert rodents (Castel and Abraham 1969; Sahni et al. 1993; Tracy and Walsberg 2001; Tirado et al. 2008; Kordonowy et al. 2017) and may help to produce some metabolic water and compensate the body water needs during WD. In the desert rodent ‘*Notomys alexis*’, 23% of body mass loss after 7 days of water deprivation was attributed to the loss of body fat. An unexpected body mass increase was observed after more than 10 days of WD in this species, which suggests that water restriction stimulated food intake, which may lead to an increased metabolic rate for water production and contribute to the maintenance of water balance (Takei et al. 2012). On the other hand, other studies performed in ‘*Meriones shawi*’, a semi-desert rodent, showed that the body mass stabilizes after three months of water restriction but without ever return to their initial values (Sahni et al. 1993; Sellami et al. 2005).

While plasma urine osmolality provides information about the body water content before, and after, water restriction, hematocrit is an indicator of plasma volume (Kutscher 1968). Decreased water intake is associated with fluid loss in both intracellular and extracellular compartments. In water-deprived Meriones, change in the extracellular compartment was reflected by increased hematocrit. The mean value of plasmatic osmolality was significantly decreased in WD Meriones for 14 and 21 days followed by an increase the last week of the experiment. In fact, the loss of free water from the extracellular fluids by evaporation and excretion can lead to an increase in plasma osmolality and a decrease in extracellular fluid volume, if uncompensated. The water-deprived Meriones in our study had increases in osmolality during the last week of the experiment, which corresponded to an increase in sodium concentration. Therefore, increases in osmolality may be due to increased concentrations of sodium but also other osmolytes.

Plasma osmolality is often unaffected in many small desert mammals in experimental conditions when they are water-deprived, which indicates that they can compensate to remain in water balance and do not become dehydrated (see Degen 1997; Heimeier et al. 2004; Heimeier and Donald 2006). Several processes have been described for maintaining body water content; including the production of metabolic water from body fat, the antidiuretic effect of AVP on urine concentration, and the extensive use of intracellular water (Horowitz and Adler 1983; Sicard 1987; Sicard 1992; Lacas et al. 2000).

### 5.2. AVP and OT expression

The present study reveals that the hypothalamic magnocellular nucleus of the desert rodent *Meriones libycus* is in a hyperactive state and shows several characteristics favoring neurosecretion. In fact, the hyperactivity of the hypothalamo-neurohypophysial system has been already described in two other gerbillidae rodents (Ouali-Hassenaoui et al. 2011). From our immunohistochemical observations, it is obvious that the hypothalamic SON of Meriones is very developed; it has a large medio-lateral extension in the dorso-lateral part of the optic chiasm. The AVP neurons are mostly located in the lateral and ventral part of this nucleus, while the OT positive neurons are essentially localized in the dorso-lateral part of the SON. In the laboratory rat, the relative location of AVP and OT neurons in the SON was initially described. Swaab et al. (1975) reported that more OT-containing cells were found in the rostral part of the SON. Vandesande and Dierickx 1975, however, described OT cells as being preferentially located in the dorsal part of the SON and VP cells in its ventral region. Another feature of heightened activity was the numerous neurons that exhibit colocalization of two neuropeptides. In comparison with the rat (Swaab et al. 1975; Armstrong 1995), the SON of our animal model was composed of a greater number of magnocellular neurons that display a high degree of immunostaining for both OT and AVP. Indeed, as shown by earlier studies in the rat, under normal basal levels of neurosecretion, few neurons present colocalization of OT and AVP while under conditions like lactation and dehydration, their incidence is significantly enhanced – up to 16% in the second day of lactation (Mezey and Kiss 1991; Telleria-Diaz et al. 2001).

After one week of WD, the immunohistochemical study showed a significant increase in vasopressin expression in the SON. These data strongly suggest that AVP is stored to be released into the blood (Ciosek 1989). This finding may be due to an increase in AVP synthesis in the SON. Many studies on desert rodents showed significant VPergic hyperactivity of the hypothalamo–neurophyseal system. In fact, AVP stores were higher in the neurohypophysis of desert rodents than in laboratory rats; the hypothalamic AVP biosynthesis was enhanced, and the releasable pool of neuropeptide was never exhausted (Bridges and James 1982; Ouali-Hassenaoui et al. 2011). Moreover, during dehydration, AVP is the first hormone to be secreted (Bouby and Fernandes 2003). Several studies have shown an increase in the bioelectrical discharge of vasopressinergic neurons of magnocellular hypothalamic nuclei during progressive dehydration (Poulain and Wakerley 1982). These data could explain and confirm that the enhanced immunoreactivity of AVP is due to an increase of peptide synthesis. Several studies in desert species have used radioimmunoassay, to examine the hormonal control of osmoregulation. In comparison to non-desert mammals, the plasma levels of AVP in desert rodents are higher than in mesic species (Castel and Abraham 1972; El-Husseini and Haggag 1974; Baddouri et al. 1984; Stallone and Braun 1988). Recent experiments using electrophysiology demonstrate that AVP neurons show a rapid increase in activity within 30 s at the onset of dry food ingestion, suggesting that the same neurons are capable of rapid bidirectional modulation of kidney function, beginning in the period prior to any systemic feedback. Indeed, this increase occurred before any significant rise in plasma osmolality which began more than 2 min after feeding onset (Bankir et al. 2017; Mandelblat-Cerf et al. 2017). This could explain the no significant changes in the plasmatic osmolality in the first week of WD in Meriones, it is now understood that thirst and AVP release are regulated not only by the classical homeostatic, intero-sensory plasma osmolality negative feedback but also by novel, extra-sensory, anticipatory signals (Bankir et al. 2017).

After four weeks of WD, we showed a decrease in AVP immunoreactivity that goes back to the same level as control animals, suggesting that AVP was released into the blood and/or in our animal model, which is an impressive way of staying hydrated. AVP is most likely released in response to extracellular fluid (ECF) hyperosmolarity in desert mammals, but many species can maintain plasma osmolarity and ECF when water-restricted and therefore suppress osmotically driven up-regulation of AVP release (Donald and Pannabecker 2015).

On the other hand, the immunohistochemical analysis has shown a decrease in OT expression in the SON after four weeks of WD. Under osmotic stimulation, previous studies suggest activation of OT synthesis and release in some experimental models (Han et al. 1992). These data could explain the increase of sodium concentration after a prolonged WD. Indeed, previous studies in rats show that OT is a natriuretic hormone that plays a fundamental role in the regulation of extracellular fluid volume (Soares et al. 1999). It was also found that several MCNs display immunoreactivity for both nonapeptides under water deprivation conditions (Jirikowski et al. 1991). It has been clearly distinguished with *in situ* PCR technique that there is some OT and VP mRNA co-expression in all of the MCNs in the rat’s SON (Xi et al. 1999). This finding reversed the concept that the expression of these peptides genes was mutually exclusive and occurs separately in the OT and AVP MCNs (Mohr et al. 1988). Under certain functional conditions, however, it has been shown that OT and AVP can be expressed in the same neuron, as determined by combined immunocytochemistry and *in situ* hybridization (Mezey and Kiss 1991). The colocalization indicates simultaneous synthesis and release of both peptides. The question of whether OT neurons are recruited into AVP expression upon prolonged osmotic stimulation, to compensate for the deficit of AVP, vasopressin neurons start to express OT or whether dormant populations of MCNs are activated to synthesize both peptides is the topic of further investigations.

In conclusion, the adaptation of rodents to life in the desert may include different combinations of morphological, physiological, and behavioral characteristics to generate mechanisms of water conservation (Bozinovic and Gallardo 2006). In *Meriones libycus*, 30 days of WD induced modifications in biological and morphofunctional parameters of the hypothalamo-neurohypophysial system’s activity. Prolonged water deprivation caused in our animal model an increase in AVP expression in the MCNs of the SON associated to a decrease of OT expression in this nucleus.

In sum, our study supports the fact that the desert rodent ‘Meriones libycus’ can survive a long period of water deprivation by physiological adaptations that reduce water loss. Beside the morphological and physiological modifications cited above, many groups of small mammals drastically reduce their energy expenditure, body temperature, metabolic rate, and water loss during torpor to avoid seasonal shortages of water or energy and to regulate the animal’s major avenues of water gain and loss (Walsberg 2000; Ehrhardt et al. 2005). However, in our species, *Meriones libycus*, a recent study reported no evidence of hypothermia or torpor (Alagaili et al. 2017), which excludes torpor as an adaptive mechanism for water saving in extreme conditions in these species.

It would be interesting to analyze several aspects of this hypothalamo-neurohypophysial system. Electronic microscopy studies can reveal the synaptic structural changes in SON magnocellular neurons with water deprivation and their physiological consequences. Furthermore, *Meriones libycus* could serve as an excellent natural model for research on osmoregulation, chronic dehydration, and metabolic syndromes.
